# From Molecular Perturbations to Functional Decline: Multi-Omics Reveals Sperm Cryodamage in Sichuan Bream (*Sinibrama taeniatus*)

**DOI:** 10.3390/ani16071014

**Published:** 2026-03-26

**Authors:** Zhe Zhao, Qilin Feng, Tianzhi Jin, Qiang Zhao, Shilin Li, Dengyue Yuan, Zhijian Wang, Fang Li

**Affiliations:** 1 Integrative Science Center of Germplasm Creation in Western China (CHONGQING) Science City, Chongqing Technology Innovation Center of Breeding, Southwest University, Chongqing 401329, China; 18883779783@163.com (Z.Z.);; 2 School of Agronomy, Xinjiang Hetian College, Hetian 848000, China; 3 Department of Aquaculture, Yibin Academy of Southwest University, Yibin 644000, China

**Keywords:** sperm cryopreservation, cryodamage, CASA, TEM, SEM, flow cytometry, proteome, metabolome, *Sinibrama taeniatus*

## Abstract

Sperm cryopreservation is pivotal for conserving fish germplasm, yet cryodamage-induced quality decline limits its application. This study focused on Sichuan bream (*Sinibrama taeniatus*), an endemic and economically important fish species in the upper Yangtze River. Based on an established cryopreservation protocol, we evaluated sperm quality using computer-assisted sperm analysis (CASA) and fertility assays, followed by a systematic assessment of structural and functional damage via flow cytometry (membrane integrity, mitochondrial potential, reactive oxygen species, and DNA fragmentation), enzymatic assays (energy metabolism and antioxidant enzymes), Western blotting, and ultrastructural observation. Finally, integrated proteomic and metabolomic analyses were employed to elucidate the underlying physiological mechanisms. The results demonstrated that freeze–thawing significantly impaired sperm motility, fertility, and ultrastructure, concurrently disrupting energy metabolism and the antioxidant system. Crucially, multi-omics revealed that these functional declines were linked to dysregulation in key pathways involving cytoskeleton organization, lipid metabolism, energy homeostasis, and oxidative stress, forming a coherent network from initial molecular perturbation to phenotypic dysfunction. This study provides a comprehensive characterization of sperm cryodamage in Sichuan bream, advancing the understanding of fish sperm cryobiology and informing targeted cryoprotection strategy development.

## Simple Summary

This study investigated the mechanisms underlying sperm cryodamage in Sichuan bream (*Sinibrama taeniatus*) to inform improved preservation strategies for its genetic resources. We found that the freeze–thaw process severely harmed sperm structure, reduced their motility, and lowered their success in fertilizing eggs. The damage was linked to problems in the sperm’s energy production, increased harmful molecules, and breakdown of their internal support system. By analyzing proteins and small chemicals inside the sperm, we identified key biological pathways disrupted by freezing. These results help explain how freezing injures fish sperm at a molecular level and provide a scientific basis for designing better freezing techniques, which will support the conservation of this species and sustainable fish farming.

## 1. Introduction

Sperm cryopreservation is a key technique in reproductive management of fish, enabling long-term storage of germplasm resources by suspending cellular metabolism in liquid nitrogen at −196 °C [1]. Since researchers first successfully froze herring sperm [2], standardized protocols have been established for more than 200 marine and freshwater fish species [3]. Sperm cryopreservation not only facilitates the synchronization of spawning cycles in seasonal breeders, thereby reducing broodstock management effort and aquaculture costs, but also supports the preservation of high-quality, endangered, or economically valuable genetic materials [4]. These advances underpin the development of germplasm banks, help maintain biodiversity, enhance breeding potential, and strengthen aquaculture’s resilience to disasters and diseases, thereby promoting sustainable industrial development.

However, the application efficiency of this technology is seriously hampered by cryodamage inevitably incurred during freezing and thawing [5]. Studies have shown that such damage is primarily triggered by extreme osmotic stress, cold shock, intracellular ice crystal formation, and dysregulation of the antioxidant defense system [6]. These factors lead to structural and functional impairments in sperm, manifested as compromised plasma membrane integrity, decreased mitochondrial membrane potential (ΔΨm), reduced metabolic enzyme activity, accumulation of reactive oxygen species (ROS), and diminished ATP levels [7,8]. DNA damage and apoptosis have also been frequently observed in thawed sperm of species such as rainbow trout (*Oncorhynchus mykiss*) [9], European sea bass (*Dicentrarchus labrax*) [10], and bocachico (*Prochilodus magdalenae*) [11]. Additionally, the freezing process may further induce epigenetic modifications (such as DNA methylation), thereby affecting gene expression and offspring development [12]. Therefore, a deeper understanding of the molecular mechanisms of cryodamage is of great significance for optimizing sperm cryopreservation strategies in fish.

Advances in omics technologies have opened new avenues for understanding the molecular basis of sperm cryodamage. In the context of cryopreservation, genomic and transcriptomic approaches have been instrumental in identifying genetic variants associated with cryotolerance and revealing gene regulatory networks during cold acclimation [13,14]. However, for mature spermatozoa, the application of these upstream omics is intrinsically limited. As highly specialized cells, sperm are characterized by a lack of bulk transcriptional and translational capacity due to extreme chromatin compaction. Consequently, the functional status of thawed sperm is determined predominantly by the stability and interaction of existing macromolecules. This renders proteomics particularly suitable for assessing their physiological status [15]. Accumulating evidence demonstrates a strong correlation between cryopreservation-induced damage and alterations in sperm protein expression, a phenomenon observed across multiple species. In human sperm, for instance, 27 differentially expressed proteins were identified after cryopreservation, primarily involved in acrosome reaction, motility, calcium homeostasis, and mitochondrial function [16]. Similarly, four common seminal plasma proteins identified across bull breeds—RNASE1-2, ACRBP, CD247, and ATP6AP2—were strongly correlated with sperm fertility, suggesting their potential as biomarkers [17]. Studies on buck sperm further suggest that the FoxO signaling pathway may alleviate cryodamage by regulating cell survival, antioxidant defense, and metabolic processes [18]. While these findings have advanced our understanding of sperm cryobiology, research on protein dynamics in cryopreserved fish sperm remains fragmented. The functional consequences of changes in protein abundance, in particular, require clarification through integrated molecular approaches to elucidate subcellular mechanisms.

Metabolites, as intermediates or end products of metabolic pathways, are integral to protein structure and function and play crucial roles in sperm physiology [19]. Metabolomics has been increasingly applied in mammalian sperm cryopreservation studies to identify associations between specific metabolites and post-thaw fertility [20,21,22]. In contrast, its application in fish sperm cryopreservation remains limited. Fish sperm possess unique biological traits: motility activates almost instantaneously upon water contact, resulting in short motility duration [23]; additionally, higher proportions of unsaturated fatty acids in plasma membranes increase susceptibility to lipid peroxidation [24]. These characteristics interact within a complex regulatory network. Integrated multi-omics approaches combining proteomics and metabolomics will be essential for systematically characterizing dynamic protein–metabolite interactions during cryopreservation and for uncovering the molecular mechanisms underlying functional maintenance in fish sperm.

Sichuan bream (*Sinibrama taeniatus*), a small economically important fish endemic to the upper Yangtze River, exhibits a typical seasonal breeding strategy characterized by asynchronous batch spawning in spring and autumn [25]. While previous studies have systematically elucidated its mechanisms of ovarian development [26,27,28], the mechanisms underlying damage during sperm cryopreservation remain unclear. Therefore, this study aims to systematically evaluate the physiological and molecular impacts of cryopreservation on Sichuan bream sperm and to uncover the underlying regulatory mechanisms. We first established a sperm cryopreservation protocol and assessed post-thaw sperm quality through motility analysis and fertility tests. The structural and functional damage to sperm was further systematically characterized using ultrastructural observation, flow cytometry, and enzyme activity assays. Additionally, proteomic and metabolomic approaches were employed to explore the associated molecular regulatory networks. The findings are expected to enhance the understanding of fish sperm cryobiology and provide a theoretical basis for optimizing germplasm preservation strategies for this species.

## 2. Materials and Methods

### 2.1. Experimental Fish and Semen Collection

Sichuan bream used in this study were laboratory-reared offspring of wild broodstock collected from the upper reaches of the Yangtze River. The fish were cultured and maintained following protocols established in our previous report [26]. Fresh sperm were collected as described previously [29]. Briefly, ten sexually mature males (body length: 85.02 ± 6.39 mm; body weight: 11.65 ± 2.56 g) in good physiological condition were selected and anesthetized with MS-222 (tricaine methanesulfonate). After drying the urogenital region, semen was collected by gentle abdominal pressure into sterile, dry, and graduated plastic tubes (approximately 0.25 mL per male). All samples were maintained at 4 °C during processing and protected from contamination by feces, urine, or blood.

### 2.2. Sperm Cryopreservation Protocol

To evaluate biological variability in cryotolerance, semen samples collected from individual males were processed and cryopreserved separately. Sperm cryopreservation was carried out using an existing research protocol [7] with minor modifications. Briefly, a two-step freezing procedure was adopted. Semen was diluted with Ringer’s solution (133.5 mM NaCl, 2.7 mM KCl, 1.9 mM CaCl_2_, 23.8 mM NaHCO_3_) at 4 °C, supplemented with 5% glycerol and 0.1 M taurine to formulate the cryoprotectant medium. The semen-to-cryoprotectant ratio was maintained at 1:9. The diluted semen was then loaded into 0.5 mL plastic straws (IMV Technologies, L’Aigle, France). After holding the samples at 4 °C for 5 min, they were placed 10 cm above liquid nitrogen for 10 min of equilibrium, then immediately immersed in liquid nitrogen for long-term storage. Following 7 days of cryopreservation, frozen samples were thawed in a 37 °C water bath for 30 s before subsequent analysis. All chemicals used were of analytical grade.

### 2.3. Computer-Assisted Sperm Analysis (CASA)

Motility analysis was performed on both fresh (*n* = 6) and cryopreserved (*n* = 6) sperm samples. To trigger sperm activation through hypoosmotic shock, samples were diluted in phosphate-buffered saline (PBS; 137 mM NaCl, 2.7 mM KCl, 10 mM Na_2_HPO_4_, 1.8 mM KH_2_PO_4_; pH 7.4; 290 mOsm/kg). Fresh sperm were diluted 1:100. For frozen-thawed sperm, a 1:10 dilution with PBS was applied to the cryopreserved samples (already diluted 1:10, see Section 2.2), resulting in a final cumulative dilution of 1:100. This ensured consistent cell concentrations (30–50 × 10^6^ cells/mL) and sufficient osmotic reduction for full activation. A 5 μL aliquot of each diluted sample was placed in a sperm counting chamber and examined under a biological microscope (DM500, Leica Microsystems, Wetzlar, Germany). Sperm motility parameters were then assessed using a computer-assisted sperm analysis system according to the manufacturer’s instructions (Zoneking, Beijing, China). Assessments were standardized to commence 10 s post activation. Spermatozoa with velocities > 5 μm/s were defined as motile. The recorded parameters included: motility percentage (MOT, %), average path velocity (VAP, μm/s), curvilinear velocity (VCL, μm/s), straight-line velocity (VSL, μm/s), amplitude of lateral head displacement (ALH, μm), beat–cross frequency (BCF, Hz), mean angular displacement (MAD, °), wobble (WOB, %), linearity (LIN, %), and straightness (STR, %). It should be noted that only fresh semen samples exhibiting sufficiently high motility (>80%) were used for cryopreservation or subsequent analyses.

### 2.4. Fertility Test

To evaluate sperm fertility, eggs were collected from ten female Sichuan bream and pooled. The eggs were fertilized separately with fresh (*n* = 6) and cryopreserved (*n* = 6) sperm at a ratio of approximately 1 × 10^7^ sperm per egg batch. After activation with fresh water, the fertilized eggs were evenly distributed in laboratory-made incubation nets and maintained at 26 °C in a recirculating aquaculture system. At 7 h post fertilization, when embryos reached the gastrula stage, fertilization rates were determined by counting the number of gastrulating embryos relative to the initial number of eggs using a stereomicroscope (SMZ25, Nikon, Tokyo, Japan). A minimum of 200 embryos were examined per sample. Dead eggs were removed, and incubation continued until 48 h post fertilization, when hatching rates were calculated as the proportion of hatched larvae relative to fertilized eggs.

### 2.5. Scanning and Transmission Electron Microscopy

Samples for scanning electron microscopy (SEM) and transmission electron microscopy (TEM) were prepared according to the existing research protocols [30] with some modifications. Fresh and frozen–thawed semen samples were centrifuged (4 °C, 200× *g*, 10 min), and the pellets were washed with PBS. The collected sperm pellets were fixed with 2.5% glutaraldehyde for 2 h at room temperature and then stored at 4 °C in the dark. After fixation, samples were rinsed three times (15 min each) with 0.1 M phosphate buffer (PB, pH 7.4) and post-fixed with 1% osmium tetroxide for 2 h at room temperature in the dark. For SEM observation, fixed samples were dehydrated through a graded ethanol series, then substituted with isoamyl acetate. Critical point drying was performed using a dryer (K850, Quorum Technologies, Laughton, UK). The dried samples were mounted on specimen stubs, sputter-coated with gold for 30 s using an ion sputter coater (MC1000, Hitachi, Tokyo, Japan), and examined under a scanning electron microscope (SU8100, Hitachi, Tokyo, Japan). For TEM analysis, fixed samples were similarly dehydrated in a graded ethanol series and embedded in epoxy resin. Ultrathin sections (60–80 nm) were cut using an ultramicrotome, collected on copper grids with supporting films, and stained with 2% uranyl acetate in alcohol followed by lead citrate. Sections were observed under a transmission electron microscope (HT7800, Hitachi, Tokyo, Japan).

### 2.6. Flow Cytometric Analysis

A BD FACS Celesta flow cytometer (BD Biosciences, San Jose, CA, USA) was employed to assess sperm membrane status (with Annexin V/PI), mitochondrial membrane potential (with JC-1), reactive oxygen species levels (with DCFH-DA), and DNA integrity (with TUNEL). For each measurement, a minimum of 100,000 sperm cells was analyzed at a flow rate of 1000 cells per second. Sperm populations were gated based on forward scatter (FSC) and side scatter (SSC) to exclude debris and aggregates. Excitation was performed using 405 nm, 488 nm, and 640 nm solid-state lasers. Green fluorescence was detected in the FITC channel (530/30 nm) and red fluorescence in the PE channel (575/25 nm). Prior to analysis, both fresh and frozen–thawed semen samples were centrifuged (4 °C, 200× *g*, 5 min), and the resulting pellets were resuspended in PBS to achieve a sperm concentration of 1 × 10^6^ cells/mL.

#### 2.6.1. Sperm Membrane Status

Sperm membrane integrity and apoptosis were assessed using an Annexin V-FITC/PI Apoptosis Detection Kit (Beyotime, Shanghai, China). Following the manufacturer’s instructions, 100 μL of sperm suspension was centrifuged (4 °C, 200× *g*, 5 min), and the pellet was resuspended in 195 μL of Annexin V-FITC binding buffer. After adding 5 μL of Annexin V-FITC and 10 μL of propidium iodide (PI) staining solution, the samples were gently mixed, incubated for 10 min at room temperature in the dark, and then immediately analyzed by flow cytometry. Annexin V-FITC binds to phosphatidylserine (PS) translocated to the outer membrane during early apoptosis, emitting green fluorescence. At the same time, PI stains DNA in necrotic cells with compromised membrane integrity, emitting red fluorescence.

#### 2.6.2. Mitochondrial Membrane Potential

Sperm mitochondrial activity was evaluated using a Mitochondrial Membrane Potential Assay Kit with JC-1 (Beyotime, China). According to the manufacturer’s protocol, 100 μL of sperm suspension was centrifuged (4 °C, 200× *g*, 5 min), and the pellet was resuspended in 500 μL of JC-1 staining solution. After incubation at 37 °C for 20 min in the dark, the samples were centrifuged (4 °C, 600× *g*, 5 min) and the supernatant was discarded. The pellet was washed twice with JC-1 staining buffer, resuspended in the same buffer, and immediately analyzed. JC-1 forms red fluorescent aggregates in mitochondria with high membrane potential.

#### 2.6.3. Reactive Oxygen Species

Intracellular ROS levels were measured using a Reactive Oxygen Species Assay Kit (Beyotime, China) with the fluorescent probe DCFH-DA. Briefly, 1 mL of sperm suspension was centrifuged (4 °C, 200× *g*, 5 min), and the pellet was resuspended in 1 mL of 10 μM DCFH-DA. After incubation at 37 °C for 20 min in the dark, the samples were immediately analyzed by flow cytometry. Intracellular ROS oxidize non-fluorescent DCFH to generate fluorescent DCF.

#### 2.6.4. DNA Fragmentation

Sperm DNA integrity was assessed using a One Step TUNEL Apoptosis Assay Kit (Beyotime, China). According to the manufacturer’s instructions, 100 μL of sperm suspension was fixed with 4% paraformaldehyde for 30 min at room temperature. After washing with PBS, the cells were permeabilized with 0.3% Triton X-100 in PBS for 5 min at room temperature. The samples were then washed twice with PBS and incubated with 50 μL of TUNEL detection solution (containing TdT enzyme and fluorescein–dUTP) at 37 °C for 60 min in the dark. Following two additional washes with PBS, the pellet was resuspended in 500 μL PBS and immediately analyzed. Sperm with DNA fragmentation (TUNEL-positive) exhibit green fluorescence.

### 2.7. Biochemical and Protein Expression Analyses of Sperm Cells

#### 2.7.1. Determination of Enzymatic Activities

Both fresh (*n* = 6) and frozen–thawed (*n* = 6) semen samples were centrifuged (4 °C, 200× *g*, 10 min). The pellets were washed with PBS and resuspended in 1 mL of PBS. Cell homogenates were prepared using an ultrasonic cell disruptor (Biosafer, Nanjing, China) with three cycles of vortexing. Enzyme activities were determined using commercial assay kits (Nanjing Jiancheng, Nanjing, China) according to the manufacturer’s instructions, measuring total protein (TP), total ATPase (T-ATP), succinate dehydrogenase (SDH), lactate dehydrogenase (LDH), superoxide dismutase (SOD), glutathione reductase (GR), and catalase (CAT). Absorbance values were measured using a microplate reader (RT-6100, Rayto, Shenzhen, China) to calculate concentration levels.

#### 2.7.2. Western Blot Analysis

Western blotting was performed on fresh (*n* = 3) and frozen–thawed (*n* = 3) sperm samples. Samples were centrifuged (4 °C, 200× *g*, 10 min), and the pellets were resuspended in PBS to achieve a standardized concentration of 1 × 10^9^ cells/mL. Aliquots containing 1 × 10^8^ spermatozoa were collected from each sample, centrifuged, and lysed in RIPA buffer supplemented with protease and phosphatase inhibitors. Following centrifugation (4 °C, 12,000× *g*, 15 min), the supernatants were collected and denatured by boiling in SDS sample loading buffer. Equal volumes of the lysates were resolved by SDS-PAGE and transferred onto polyvinylidene difluoride (PVDF) membranes. The membranes were blocked with 5% non-fat milk in TBST for 1 h at room temperature and then incubated overnight at 4 °C with primary antibodies (all from Boster Bio, Pleasanton, CA, USA): anti-SOD1 (AZO73872), anti-SOD2 (AZQ6P980), anti-CAT (AZQ9PT92), anti-GPX5 (A10324-1), and anti-α-Tubulin (AZQ6NWK7), which served as the loading control. After washing with TBST, the membranes were incubated with horseradish peroxidase (HRP)-conjugated secondary antibodies for 1 h at room temperature. Protein bands were visualized using an enhanced chemiluminescence (ECL) kit and captured with a ChemiDoc imaging system (Bio-Rad, Hercules, CA, USA). Quantitative densitometric analysis was conducted using ImageJ software (version 1.53, National Institutes of Health, Bethesda, MD, USA), with the relative expression levels of target proteins calculated as the ratio of their band intensities to that of α-Tubulin.

### 2.8. Data-Independent Acquisition (DIA) Proteomics Investigation

#### 2.8.1. Protein Extraction

Given the limited biomass of individual samples, fresh and frozen–thawed sperm samples were pooled within their respective groups. Three replicates were processed from these pools to ensure technical reproducibility and sufficient protein yield. Semen samples from three fresh and three post-thaw specimens were centrifuged (4 °C, 200× *g*, 10 min) to remove seminal plasma. The resulting pellets were washed with PBS, and approximately 30 mg of precipitate was collected and stored at –80 °C for subsequent protein extraction. For protein extraction, an appropriate volume of lysis buffer (8 M urea + 1% SDS) supplemented with protease inhibitor (Bimake, Houston, TX, USA) was added to each sample. The mixtures were sonicated on ice for 2 min, followed by centrifugation at 12,000× *g* for 20 min at 4 °C. Protein concentration in the supernatant was determined using a protein analyzer (M5350AA, Agilent Technologies, Santa Clara, CA, USA) with 1 μL of the extract.

Protein samples (100 μg) were resuspended in 100 mM triethylammonium bicarbonate (TEAB) buffer. The resuspended proteins were reduced with 10 mM tris (2-carboxyethyl) phosphine (TCEP) at 37 °C for 60 min, followed by alkylation with 40 mM iodoacetamide (IAM) at room temperature for 40 min in the dark. After centrifugation at 10,000× *g* and 4 °C for 20 min, the resulting pellet was collected and reconstituted in 100 μL of 100 mM TEAB buffer. Trypsin digestion was then performed at a 1:50 (*w*/*w*) enzyme-to-protein ratio, with incubation at 37 °C overnight. Following trypsin digestion, peptides were dried under vacuum and subsequently reconstituted in 0.1% trifluoroacetic acid (TFA). Desalting was performed using HLB cartridges, after which the peptides were again concentrated by vacuum drying. Peptide quantification was carried out using a NANO DROP ONE spectrophotometer (Thermo Scientific, Waltham, MA, USA) based on UV absorbance measurements.

#### 2.8.2. DIA Mass Spectrometry Analysis

Based on the quantification results, peptide samples were analyzed using a Vanquish Neo UHPLC system coupled to an Orbitrap Astral mass spectrometer (Thermo Scientific) at Majorbio Bio-Pharm Technology Co., Ltd. (Shanghai, China). Separation was performed on a uPAC High Throughput column (75 μm × 5.5 cm, Thermo Scientific) with mobile phase A (2% acetonitrile, 0.1% formic acid in water) and mobile phase B (80% acetonitrile, 0.1% formic acid in water). The chromatographic separation was conducted over 8 min. Data-independent acquisition (DIA) was performed with a mass scanning range of 100–1700 *m*/*z*.

#### 2.8.3. Protein Identification and Quantification

DIA raw data were processed using Spectronaut software (version 19) with the following parameters: peptide length range 7–52 amino acids; enzyme specificity set to trypsin/P; maximum of two missed cleavages allowed; fixed modification of carbamidomethylation on cysteine; variable modifications including methionine oxidation and protein N-terminal acetylation. False discovery rates (FDR) were set to ≤0.01 for both proteins and peptides, with peptide confidence threshold ≥ 99% and XIC width tolerance ≤ 75 ppm. Protein quantification was performed using the MaxLFQ algorithm.

### 2.9. Untargeted Metabolomic Investigation

#### 2.9.1. Metabolite Extraction

For metabolomic analysis, aliquots from the same pooled samples used for proteomics were processed. Both fresh (*n* = 6) and frozen-thawed (*n* = 6) semen samples were centrifuged (4 °C, 200× *g*, 10 min) to remove seminal plasma. After washing with PBS, 50 mg of pellet was collected and stored at −80 °C for subsequent metabolite extraction. For each group, three biological replicates, as used in the proteomic analysis, were selected for proteomic-metabolomic association studies. To each sample, 800 μL of extraction solution (methanol:acetonitrile:water = 2:2:1, *v*/*v*/*v*, containing an isotopically labeled internal standard mixture) was added. The samples were homogenized using a frozen tissue grinder for 6 min (−10 °C, 50 Hz), followed by low-temperature ultrasonic extraction for 30 min (5 °C, 40 kHz) and incubation at −20 °C for 30 min. Subsequently, the samples were centrifuged (4 °C, 13,000× *g*, 10 min), and the supernatant was collected for further analysis. Quality control (QC) samples were prepared by pooling 10 μL of each sample’s extract to evaluate the accuracy and reproducibility of the LC-MS system.

#### 2.9.2. LC-MS/MS Analysis and Data Processing

LC-MS/MS analysis was performed using a UHPLC system (Vanquish, Thermo Fisher Scientific) coupled with an Orbitrap Exploris 120 mass spectrometer (Thermo). Separation was achieved on a UPLC BEH Amide column (2.1 mm × 100 mm, 1.7 μm) with a mobile phase consisting of 25 mmol/L ammonium acetate and 25 mmol/L ammonium hydroxide in water (pH 9.75) (A) and acetonitrile (B). The autosampler temperature was maintained at 4 °C with an injection volume of 2 μL. The mass spectrometer operated in information-dependent acquisition (IDA) mode controlled by Xcalibur software (version 4.1, Thermo Fisher Scientific, Waltham, MA, USA). ESI source conditions were set as follows: sheath gas flow 50 arb, auxiliary gas flow 15 arb, capillary temperature 320 °C, full MS resolution 60,000, MS/MS resolution 15,000, collision energy 10/30/60 eV in NCE mode, and spray voltage 3.8 kV (positive) or −3.4 kV (negative).

Raw data were converted to mzXML format using ProteoWizard (version 3.0, Palo Alto, CA, USA) and processed with an in-house R-based pipeline incorporating XCMS for peak detection, alignment, and integration. Metabolite annotation was performed against an internal MS2 database (BiotreeDB) with an annotation cutoff score of 0.3. Compounds identified in positive (POS) and negative (NEG) modes were consolidated prior to subsequent analysis.

#### 2.9.3. Integrated Proteomic–Metabolomic Analysis

Differential expression data from proteomics and metabolomics were integrated using the DIABLO framework from the mixOmics package (version 6.22.0) in R software (version 4.2.0, R Foundation for Statistical Computing, Vienna, Austria). Multiblock sparse Partial Least Squares Discriminant Analysis (sPLS-DA) was employed to construct model components. Pearson correlation coefficients were calculated to quantify associations between metabolites and proteins within each group, with visualization implemented in Python 3.9.19. Shared KEGG pathways between proteomic and metabolomic datasets were identified for subsequent functional interpretation.

### 2.10. Statistical and Bioinformatic Analysis

For functional assays (CASA, fertility test, flow cytometric and cell biochemical analyses, *n* = 6) and Western blot analysis (*n* = 3), data are expressed as mean ± standard deviation (SD). Prior to analysis, data normality was verified using the Shapiro–Wilk test. Comparisons between fresh and frozen–thawed groups were conducted using a paired Student’s *t*-test, with a *p*-value < 0.05 considered statistically significant. Statistical analyses were performed using SPSS 23.0 (SPSS Inc., Chicago, IL, USA).

For proteomic analysis (*n* = 3), differentially abundant proteins (DAPs) between experimental groups were identified using the “*t*-test” package in R (version 4.2.0), with significance thresholds set at fold change (FC) > 2 or < 0.5 and *p*-value < 0.05. Functional annotation of sequences was performed using the EggNOG, Gene Ontology (GO), Kyoto Encyclopedia of Genes and Genomes (KEGG), and Pfam databases. Subcellular localization was predicted using the WOLF PSORT online tool (https://www.genscript.com/wolf-psort.html, accessed on 2 September 2025). Subsequently, DAPs were subjected to GO and KEGG enrichment analyses. Protein–protein interaction (PPI) networks were constructed using the STRING database (version 11.5; https://string-db.org/) with a confidence score threshold > 0.7, and the resulting networks were visualized using Cytoscape software (version 3.9.1).

For metabolomic analysis (*n* = 6), processed data were imported into SIMCA 16 (Umetrics, Umeå, Sweden) for principal component analysis (PCA) and orthogonal projections to latent structure-discriminant analysis (OPLS-DA). Model quality was evaluated using R^2^Y (Goodness of Fit) and Q^2^ (Predictive Ability) metrics. Metabolites with variable importance in projection (VIP) > 1 and *p*-value < 0.05 were considered significantly different. Differential metabolites (DAMs) were subjected to KEGG pathway analysis using MetaboAnalyst (www.metaboanalyst.ca).

## 3. Results

### 3.1. Sperm Quality Assessment: Motility and Fertility

To evaluate the quality of cryopreserved Sichuan bream sperm, cryopreserved samples were thawed after 7 days of storage and immediately subjected to computer-assisted sperm analysis (CASA). The kinematic parameters are summarized in Table 1. Post-thaw sperm motility (MOT) decreased to 39.59 ± 7.81%, approximately half of that observed in fresh sperm (82.22 ± 1.92%). Except for the mean angular displacement (MAD) and beat–cross frequency (BCF), all other sperm kinematic parameters showed statistically significant reductions (*p* < 0.01) following cryopreservation.

**Figure 1:**
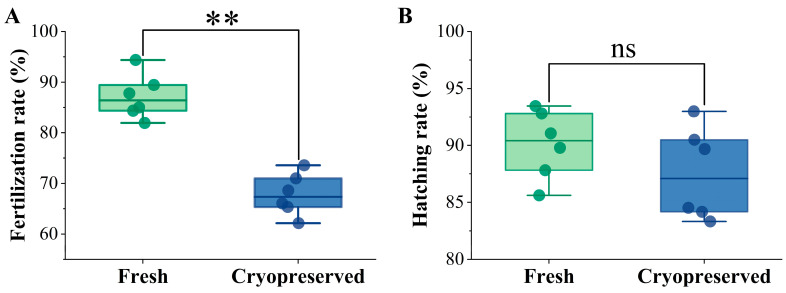
Fertilization rate (**A**) and hatching rate (**B**) of fresh and cryopreserved sperm. **: *p* < 0.01; ^ns^: not significant (*p* > 0.05).

Fertilization success was determined by the proportion of eggs developing to the gastrula stage at 7 h post fertilization. The fertilization rate for fresh sperm was 87.16 ± 4.02%, whereas a significant decrease to 67.79 ± 3.76% was observed in the cryopreserved group (*p* < 0.05) (Figure 1A). The hatching rate of cryopreserved sperm (88.37 ± 4.54%) was lower than that of fresh sperm (90.09 ± 2.73%), but the difference was not statistically significant (*p* > 0.05) (Figure 1B).

### 3.2. Ultrastructural Observations of Sperm Cells

**Figure 2:**
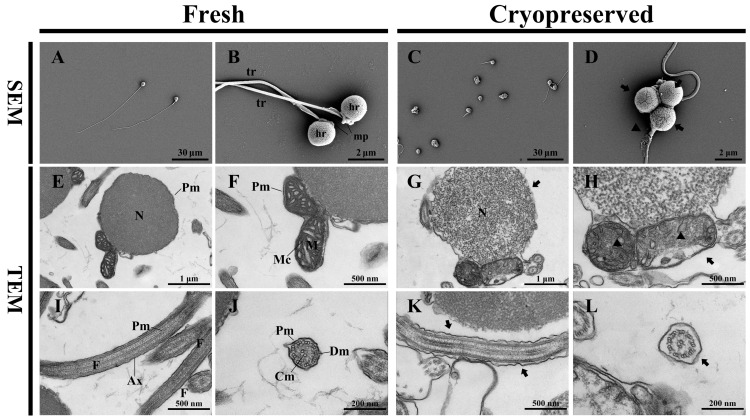
Ultrastructure of fresh and cryopreserved sperm. (**A**) Fresh sperm showing intact overall morphology. (**B**) Detailed view of fresh sperm structures, hr: head region, mp: midpiece, tr: tail region. (**C**) Cryopreserved sperm exhibiting shrinkage and structural loss. (**D**) Localized magnification of cryopreserved sperm, arrows: plasma membrane rupture, triangles: flagellar fragmentation. (**E**) Head of fresh sperm with intact plasma membrane (Pm) and densely packed nucleus (N). (**F**) Mitochondria (M) in fresh sperm displaying well-defined mitochondrial cristae (Mc) and intact double membranes, Pm: plasma membrane. (**G**) Head of cryopreserved sperm showing disrupted plasma membrane and chromatin leakage, arrows: membrane rupture, N: nucleus. (**H**) Impaired mitochondria in cryopreserved sperm, arrows: membrane rupture, triangles: degenerated cristae. (**I**) Tail region of fresh sperm with intact flagellum (F), plasma membrane (Pm), and axoneme (Ax). (**J**) Cross-section of fresh sperm flagellum, Pm: plasma membrane, Dm: doublet microtubules, Cm: central microtubules. (**K**) Cryopreserved sperm flagellum showing separation of the plasma membrane from the axoneme, arrows: enlarged interstitial spaces. (**L**) Cross-section of cryopreserved sperm flagellum, arrows: expanded interstitial spaces.

SEM analysis revealed that fresh spermatozoa of Sichuan bream consisted of three distinct regions: the head, midpiece, and flagellum (Figure 2A). The head exhibited an ovoid shape and lacked an acrosome. The midpiece appeared as a small spherical structure attached to the basal region of the head, connecting the nuclear membrane to the flagellum. The flagellum was slender and elongated (Figure 2B). After freeze–thaw cycling, sperm morphology showed significant alterations, including shrinkage, structural loss, and deformation (Figure 2C). Specific damage included rupture of the plasma membrane in the head region and fragmentation of the flagellum (Figure 2D). TEM observations demonstrated that a densely packed nucleus predominantly occupied the head of fresh sperm with high electron density. Both the plasma membrane and nuclear envelope exhibited an undulating, irregular surface (Figure 2E). The midpiece contained well-organized mitochondria with clearly visible cristae and intact double membranes (Figure 2F). The flagellum consisted of an axoneme surrounded by a plasma membrane, extending throughout the length of the tail (Figure 2I). A cross-sectional image confirmed the typical “9 + 2” microtubule arrangement, comprising nine peripheral doublet microtubules (DM) and two central singlet microtubules (CM) (Figure 2J).

Following cryopreservation, ultrastructural analysis revealed extensive damage. The sperm head showed extensive plasma membrane disruption and chromatin leakage, accompanied by markedly reduced electron density (Figure 2G). Mitochondria in the midpiece appeared swollen and disrupted, with dissolved membranes and degenerated cristae (Figure 2H). The flagellum exhibited structural loosening, characterized by the detachment of the plasma membrane from the axoneme, resulting in enlarged sub-membranous spaces (Figure 2K). Although the “9 + 2” microtubule arrangement remained largely intact without apparent dissociation of central microtubules, cross-sections revealed noticeably increased interstitial spaces (Figure 2L).

### 3.3. Flow Cytometric Analysis of Sperm Physiological Status

**Figure 3:**
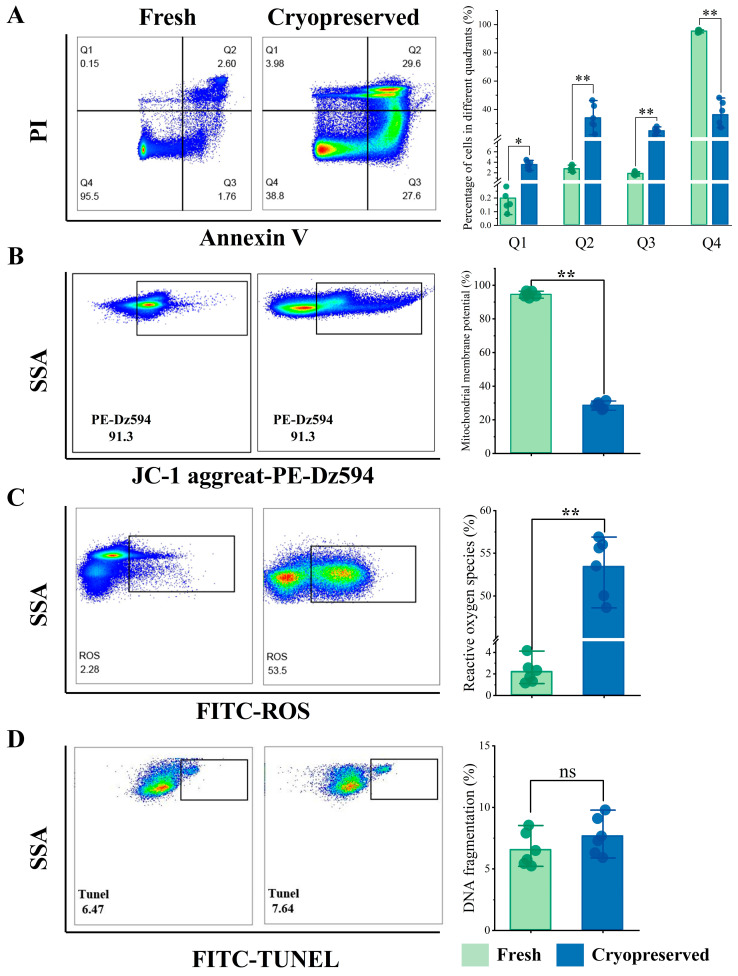
Flow cytometric analysis of sperm physiological status. (**A**) Representative scatter plots of Annexin V-FITC/PI staining in fresh and cryopreserved sperm, with proportions of cells in different quadrants. Q4: viable cells (Annexin V^−^/PI^−^); Q3: early apoptotic cells with phosphatidylserine externalization (Annexin V^+^/PI^−^); Q2: late apoptotic cells with membrane integrity loss (Annexin V^+^/PI^+^); Q1: detection within permissible error limits (Annexin V^−^/PI^+^). (**B**) JC-1 staining profiles and proportions of sperm with high ΔΨm. (**C**) ROS detection profiles and proportions of sperm with elevated ROS levels. (**D**) TUNEL assay profiles and proportions of sperm with DNA fragmentation. **: *p* < 0.01; *: *p* < 0.05; ^ns^: not significant (*p* > 0.05).

Quadrant analysis of Annexin V/PI dual staining identified four distinct sperm subpopulations (Figure 3A). Compared to fresh sperm, cryopreserved samples exhibited a highly significant reduction in viable cell population (Q4) (*p* < 0.01). The proportions of early apoptotic sperm with phosphatidylserine externalization (Q3, 24.55 ± 1.86%) and late apoptotic sperm with membrane integrity loss (Q2, 33.73 ± 8.20%) both demonstrated statistically significant increases following cryopreservation (*p* < 0.01). Evaluation of mitochondrial membrane potential (ΔΨm) using JC-1 staining (Figure 3B) revealed that the percentage of sperm with high ΔΨm decreased markedly from 94.48 ± 1.54% in fresh samples to 28.38 ± 1.91% after cryopreservation (*p* < 0.01). Similarly, ROS levels showed substantial differences between groups (Figure 3C), with significantly higher ROS generation observed in cryopreserved sperm (*p* < 0.01). In contrast, the TUNEL assay detected no statistically significant difference in DNA fragmentation between fresh and cryopreserved sperm (*p* > 0.05) (Figure 3D).

### 3.4. Biochemical and Protein Expression Analysis of Sperm Cells

**Figure 4:**
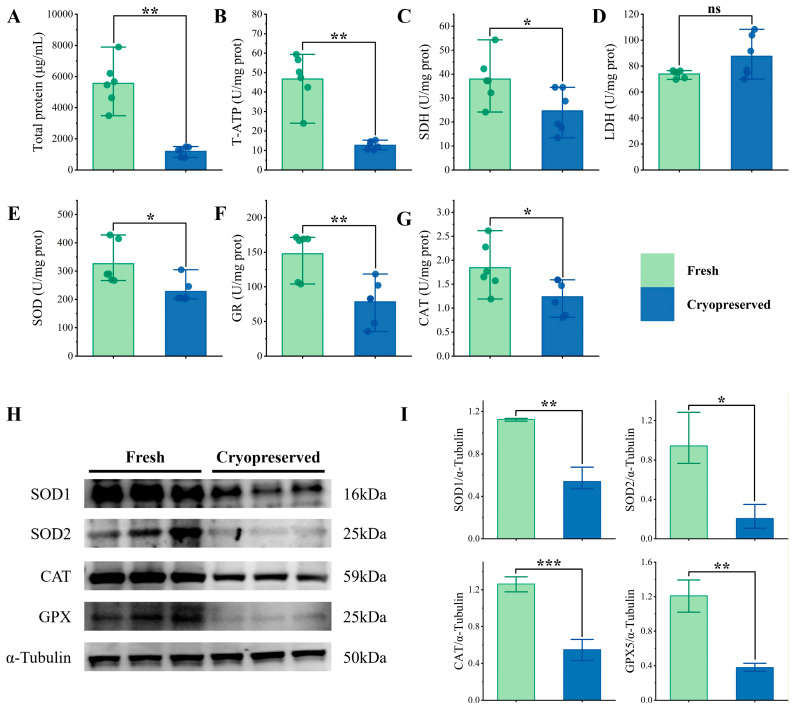
Effects of cryopreservation on key biochemical parameters and antioxidant protein expression of sperm. (**A**) Total protein. (**B**) T-ATP. (**C**) SDH. (**D**) LDH. (**E**) SOD. (**F**) GR. (**G**) CAT. (**H**) Representative Western blot images of SOD1, SOD2, CAT, and GPX5 in fresh and cryopreserved sperm. (**I**) Quantitative densitometric analysis of protein expression. The relative protein levels are expressed as the ratio of the target protein band intensity to that of the internal control, α-Tubulin. ***: *p* < 0.001; **: *p* < 0.01; *: *p* < 0.05; ^ns^: not significant (*p* > 0.05).

We evaluated the levels of total protein (TP), energy metabolism enzymes—total ATPase (T-ATP), succinate dehydrogenase (SDH), and lactate dehydrogenase (LDH)—as well as antioxidant enzymes including superoxide dismutase (SOD), glutathione reductase (GR), and catalase (CAT) in both fresh and cryopreserved sperm. As shown in Figure 4A–G, cryopreservation led to a significant reduction in TP content (*p* < 0.01). Among energy metabolism enzymes, LDH activity showed a slight but statistically non-significant increase in cryopreserved sperm (*p* > 0.05). In contrast, T-ATP and SDH activities were significantly lower than those in fresh samples (*p* < 0.05). Furthermore, the activities of all assessed antioxidant enzymes—SOD, CAT, and GR—were significantly decreased in cryopreserved sperm compared to fresh controls (*p* < 0.05).

In order to elucidate the molecular basis of this impaired antioxidant capacity, the protein expression levels of key antioxidant enzymes were examined via Western blot (Figure 4H). The results showed that the protein abundances of SOD1, SOD2, CAT, and GPX5 were all significantly reduced in cryopreserved sperm compared to fresh controls (Figure 4I), corroborating the decreased enzymatic activities measured by biochemical assays. This consistency between activity and protein expression data strengthens the conclusion that cryopreservation compromises the antioxidant defense system through downregulation of core antioxidant enzymes at the protein level.

### 3.5. Proteomics Information Based on DIA

**Figure 5:**
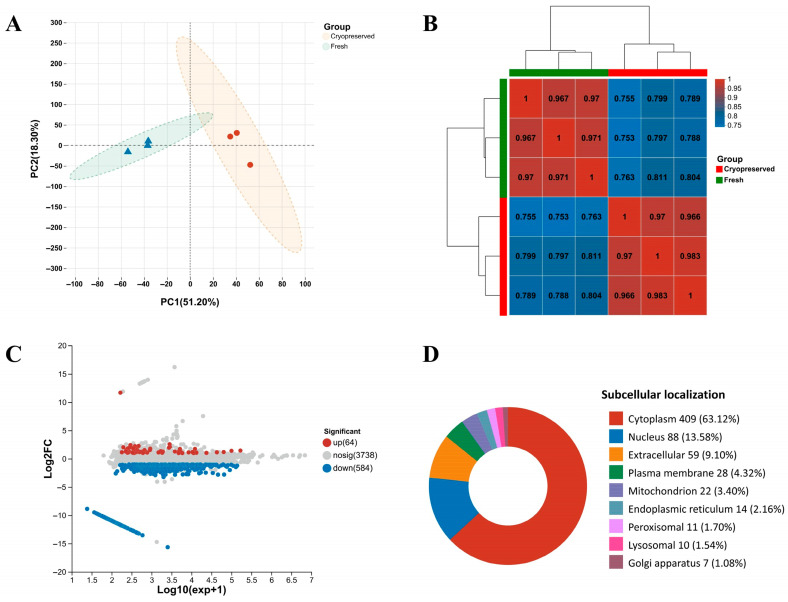
Proteomic profiles of fresh and cryopreserved sperm. (**A**) Principal component analysis. (**B**) Pearson correlation analysis of protein abundance profiles among biological replicates. The numbers inside the squares indicate the Pearson correlation coefficients between samples. (**C**) Number of DAPs between groups. (**D**) Subcellular localization of DAPs.

To further investigate the molecular impact of cryopreservation on Sichuan bream sperm, we conducted a data-independent acquisition (DIA) proteomic analysis. A total of 50,595 unique peptides corresponding to 4598 proteins were identified, among which 4448 proteins were quantitatively analyzed across all samples (Supplementary Figure S1). Principal component analysis (Figure 5A) and Pearson correlation analysis (Figure 5B) demonstrated strong intra-group reproducibility and clear inter-group separation. Using thresholds of fold change > 2 or <0.5 with *p* < 0.05, we identified 648 differentially abundant proteins (DAPs) in cryopreserved sperm compared to fresh controls, including 64 upregulated and 584 downregulated proteins (Figure 5C). The complete DAPs list is in Supplementary Table S1. Subcellular localization prediction revealed that DAPs were primarily distributed in the cytoplasm (63.12%), nucleus (13.58%), and extracellular regions (9.10%) (Figure 5D).

**Figure 6:**
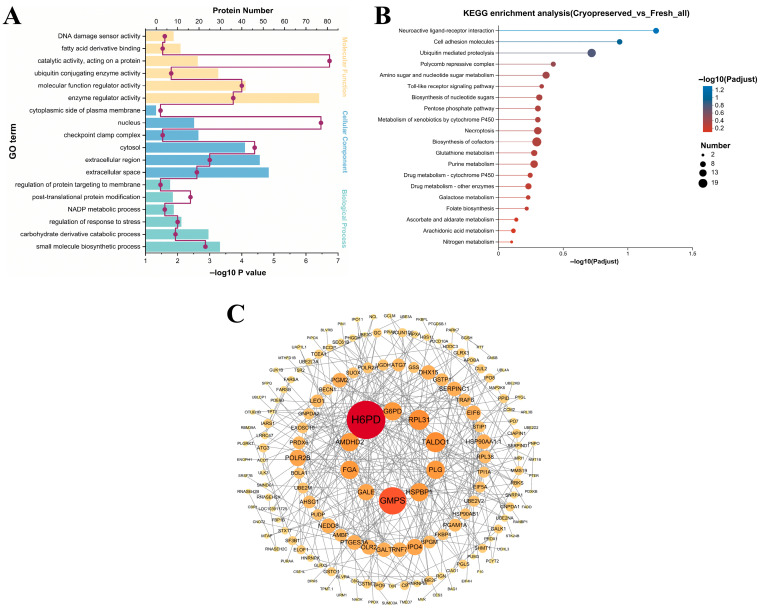
Functional analysis of DAPs. (**A**) GO enrichment analysis. (**B**) KEGG pathway enrichment analysis. (**C**) PPI network.

Functional characterization of DAPs was performed through Gene Ontology (GO) enrichment analysis, categorized into three domains: biological process (BP), cellular component (CC), and molecular function (MF). As shown in Figure 6A, downregulated DAPs in cryopreserved sperm were primarily enriched in MF terms such as enzyme regulator activity, molecular function regulator activity, and fatty acid derivative binding. Within the CC domain, significant enrichment was observed in extracellular regions, cytoplasm, and nucleus. For BP, DAPs were mainly associated with the regulation of response to stress, NADP metabolic process, and post-translational protein modification. KEGG pathway analysis revealed the top 20 enriched pathways (Figure 6B). Crucially, metabolic pathways such as the pentose phosphate pathway, glutathione metabolism, arachidonic acid metabolism, polycomb repressive complex, and ubiquitin-mediated proteolysis were significantly suppressed in cryopreserved sperm compared to fresh controls. Complete GO and KEGG enrichment results are provided in Supplementary Tables S2 and S3, respectively.

Protein–protein interaction (PPI) networks were constructed using the STRING database (v11.5) with confidence scores > 0.7. After removing self-loops and disconnected proteins, the resulting network contained 168 nodes and 299 edges (Figure 6C). The top 10 highly connected hub proteins included H6PD, GMPS, RPL31, TALDO1, FGA, PLG, AMDHD2, HSPBP1, G6PD, and GALE.

### 3.6. Untargeted Metabolomic Profiling

**Figure 7:**
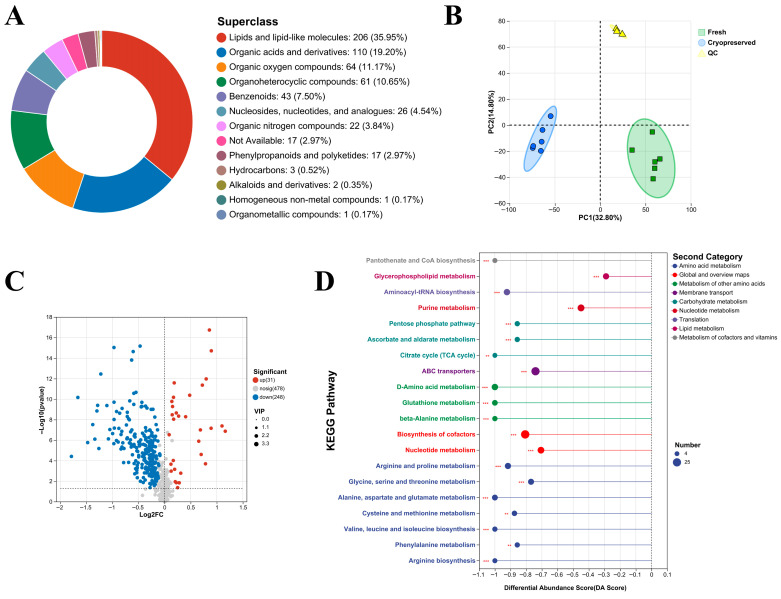
Metabolomic profiles of fresh and cryopreserved sperm. (**A**) Superclass classification of metabolites; (**B**) Principal component analysis; (**C**) Number of DAMs between groups; (**D**) Differential abundance score plot of KEGG pathway enrichment analysis showing predominantly suppressed pathways in cryopreserved sperm. ***: *p* < 0.001; **: *p* < 0.01.

We employed untargeted metabolomics to compare the metabolic profiles of fresh and cryopreserved sperm from Sichuan bream. After merging data from positive and negative ionization modes, 10,296 valid peaks were obtained, corresponding to 757 identified metabolites (Supplementary Table S4). Superclass classification using the HMDB database revealed that lipids and lipid-like molecules, organic acids and derivatives, and organic oxygen compounds constituted the predominant categories (Figure 7A).

Principal component analysis (PCA) and orthogonal partial least squares-discriminant analysis (OPLS-DA) were used to evaluate metabolic differences. PCA showed clear separation between fresh and cryopreserved sperm samples (Figure 7B). OPLS-DA further demonstrated distinct metabolic clustering between groups, with R^2^Y and Q^2^ values indicating model quality (Supplementary Figure S2).

Using variable importance in projection (VIP) > 1 from OPLS-DA and *p* < 0.05 from Student’s *t*-test as thresholds, we identified 279 differentially abundant metabolites (DAMs) in the comparison between fresh and cryopreserved sperm, including 31 upregulated and 248 downregulated metabolites (Figure 7C). Detailed information on DAMs is provided in Supplementary Table S5. KEGG enrichment analysis of DAMs, visualized using the differential abundance (DA) score, revealed that the top 20 enriched pathways were predominantly downregulated (Figure 7D). Cryopreservation significantly suppressed pathways involved in carbohydrate metabolism (e.g., TCA cycle, pentose phosphate pathway), lipid metabolism (e.g., glycerophospholipid metabolism), and amino acid metabolism (e.g., phenylalanine metabolism, glutathione metabolism). Complete KEGG enrichment results are provided in Supplementary Table S6.

### 3.7. Correlation Analysis of DAPs and DAMs

**Figure 8:**
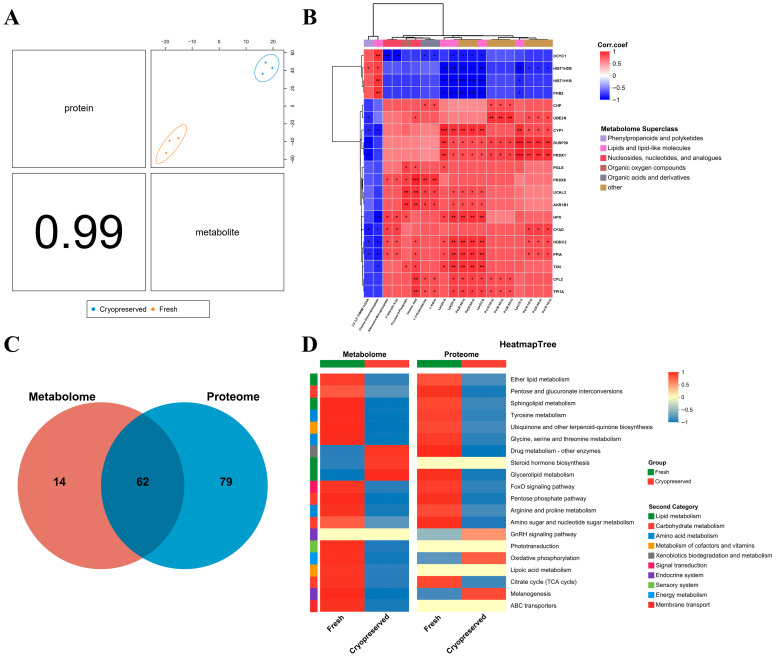
Integrated analysis of DAPs and DAMs. (**A**) Overall concordance between proteomic and metabolomic datasets; (**B**) Correlation heatmap of DAPs and DAMs; (**C**) Venn diagram showing numbers of KEGG pathways shared or unique to DAPs and DAMs; (**D**) Abundance changes to DAPs and DAMs in shared KEGG pathways between groups. ***: *p* < 0.001; **: *p* < 0.01; *: *p* < 0.05.

The DIABLO model showed strong overall consistency between proteomic and metabolomic datasets (Figure 8A). Correlation analysis revealed many-to-many interactions between proteins and metabolites, forming a complex regulatory network (Figure 8B). Complete correlation data are provided in Supplementary Table S7. After independent KEGG annotation of DAPs and DAMs, 62 pathways were found to be shared between the two omics datasets (Figure 8C). Notably, numerous proteins and metabolites exhibited coordinated reductions in abundance in cryopreserved sperm (Figure 8D), particularly in lipid metabolism pathways including sphingolipid metabolism and glycerolipid metabolism; carbohydrate metabolism pathways such as pentose and glucuronate interconversions, the pentose phosphate pathway, and the TCA cycle; as well as energy metabolism pathways including oxidative phosphorylation.

## 4. Discussion

Cryopreservation of sperm is a pivotal technology for germplasm resource conservation and large-scale aquaculture [31]. However, its application efficiency is significantly constrained by cryo-induced damage occurring during the freezing–thawing process. Although previous studies have preliminarily characterized the decline in post-thaw sperm quality using morphological and physiological indicators [32,33], the underlying molecular network driving cellular injury remains incompletely understood. In this study, we confirmed that cryopreserved sperm of Sichuan bream exhibited significantly impaired motility and fertilizing capacity. Further analysis demonstrated substantial damage to cellular ultrastructure and physiological functions, including structural abnormalities in the plasma membrane, mitochondria, and flagellum, accompanied by decreased activities of key energy metabolism enzymes and induction of oxidative stress. Proteomic and metabolomic profiling revealed hundreds of differentially expressed molecules and identified coregulated pathways, including lipid metabolism, carbohydrate metabolism, and energy metabolism. This paper will discuss these findings to elucidate the molecular logic of cryodamage, offering new perspectives on fish sperm cryobiology and optimizing cryopreservation protocols.

### 4.1. Cytoskeletal Collapse and Membrane Disintegration Are the Basis of Structural Damage

Ultrastructural examination revealed extensive morphological damage in Sichuan bream sperm following cryopreservation, including plasma membrane rupture, chromatin loss, mitochondrial cristae disappearance, and abnormal flagellar structure. These structural injuries are consistent with previously reported patterns of cryodamage and are considered the physical basis for the loss of sperm function. For instance, similar plasma membrane blebbing and mitochondrial swelling have been extensively documented in other cyprinids like common carp (*Cyprinus carpio*) [34] and marine species such as Atlantic croaker (*Micropogonias undulatus*) [35], suggesting a conserved physical mechanism of cryodamage across teleosts. However, the molecular mechanisms driving these changes remain a key scientific question that has not been fully elucidated.

The actin cytoskeleton plays a critical role in maintaining sperm morphology, regulating cell volume, and coordinating motility [36]. During freezing, sperm undergo cold shock and osmotic fluctuations, and cytoskeletal proteins—being highly sensitive to temperature and osmotic changes—may represent early targets of cryodamage [37]. In this study, we detected significant downregulation of several cytoskeleton-associated proteins (WASL, ARPC5L, ACTR1B, CFL2, WDR1, and ACTN1). Among them, WASL, a core activator of the Arp2/3 complex, has decreased expression, potentially weakening the complex’s activation ability [38]. Further downregulation of intrinsic subunits ARPC5L and ACTR1B may compromise complex stability and nucleation efficiency, potentially leading to defective actin network assembly [39,40,41]. Moreover, reduced expression of CFL2 and WDR1 collectively likely impeded actin filament depolymerization, which could result in stalled cytoskeletal turnover and diminished plasticity for environmental adaptation [42,43]. Downregulation of ACTN1 might prevent effective cross-linking of nascent actin filaments, possibly leading to failed stress fiber assembly and reduced mechanical strength and pressure resistance of the plasma membrane [44]. Overall, the combined decline in the expression levels of these molecules in cryopreserved sperm may have exacerbated the functional impairment of the actin cytoskeleton in maintaining its structure.

The flagellum, as the core motile structure of sperm, relies on precise regulation of microtubules [45]. The manchette structure, composed of α- and β-tubulin, guides tail elongation [46]. This study identified a significant post-thaw decrease in STK33, which has been shown to interact with α-tubulin to organize manchette microtubules; its downregulation may thus contribute to structural abnormalities and tail deformities [47]. Furthermore, a comprehensive downregulation of the microtubule-folding chaperones TBCD and TBCE was detected in cryopreserved sperm. Given their role in promoting the correct folding of α/β-tubulin heterodimers, these changes are likely to cause structural defects and reduced stability of the axonemal microtubules [48,49], and to some extent explain why sperm flagella are more prone to breakage after thawing.

Notably, the decline in sperm motility after cryopreservation is not only associated with structural proteins but also closely linked to potential functional impairment of motility-related proteins [50]. In cryopreserved sperm, we observed a significant downregulation of CFAP300, a key component of the central microtubule complex of the axoneme. The reduction in CFAP300 abundance may alter the symmetry of flagellar beating, potentially causing a switch from forward movement to in situ rotation of sperm [51,52], which is consistent with the results observed in this study, including an increase in mean angular displacement (MAD), a disorder in beat–cross frequency (BCF), and a comprehensive decline in forward movement parameters. Together, these mechanisms may contribute to the severe impairment of sperm motility after freezing–thawing, ultimately affecting fertilizing potential.

Sperm membrane stability is closely linked to its structure and function. Membrane integrity directly affects sperm motility, capacitation, and fertilizing ability, serving as a fundamental determinant of reproductive success [53]. Lipids, as major membrane constituents, play a central role in maintaining structural integrity, regulating fluidity, and facilitating signal transduction [54]. Phospholipids are the most abundant lipid class in sperm membranes, predominantly glycerophospholipids (GPLs) such as phosphatidylcholine (PC), phosphatidylethanolamine (PE), phosphatidylinositol (PI), and phosphatidylserine (PS), which exhibit asymmetric distribution across membrane leaflets [55,56,57]. Externalization of PS from the inner to the outer leaflet is an early marker of membrane damage and apoptosis, detectable via Annexin V staining. In this study, flow cytometry showed a significant increase in Annexin V^+^ sperm after cryopreservation, indicating severe membrane disruption and compromised cell survival.

Differential metabolite analysis further revealed a marked downregulation of multiple PC and PE species in cryopreserved sperm, suggesting substantial alterations in membrane lipid composition. From a biosynthetic perspective, the common precursors of GPLs, glycerol and glycerol-3-phosphate, were both significantly reduced, likely restricting the synthesis of PC and PE at the source [58,59]. Within the Kennedy pathway, key substrates, including cytidine, choline, and intermediates such as phosphocholine and phosphoethanolamine, were also detected to be comprehensively downregulated. Proteomic results further indicated decreased expression of rate-limiting enzymes PCYT1A and PCYT2 in cryopreserved sperm, collectively suggesting impaired PC and PE biosynthesis. Notably, PE is particularly abundant in mitochondrial membranes, where it helps maintain inner membrane structure and cristae morphology [60]. Thus, impaired PC and PE synthesis may not only compromise plasma membrane integrity but also disrupt mitochondrial function, contributing to energy metabolism dysfunction.

Cholesterol and its ratio to phospholipids critically regulate membrane fluidity, permeability, and phase transition behavior, serving as key determinants of membrane stability [53]. This study detected significant downregulation of the cholesterol transport protein NPC2, which plays an important role in cholesterol homeostasis. Its dysregulation has been linked to membrane structural damage and impaired fertilizing capacity [61], suggesting that its decreased expression may be a marker of progressive membrane deterioration. Loss of membrane integrity is typically accompanied by intracellular protein leakage. Consistently, we observed a decrease in total protein levels after freezing-thawing, aligning with previous findings in rainbow trout [37] and common carp [62]. Concurrently, upregulation of ribosomal protein RPL31 may reflect a compensatory mechanism activated in response to protein loss.

Furthermore, the heat shock protein system was significantly altered after cryopreservation, with decreased expression of HSP70, HSP90A, HSP90, and the chaperone HSPBP1. HSPBP1was identified as a hub protein in the cryo-response. As a key co-chaperone of HSP70, it regulates HSP70-mediated protein folding, transport, and degradation, playing a central role in cold stress response [63,64]. Previous studies have shown that HSP70 and HSP90 expression correlate positively with sperm motility, morphology, and freeze tolerance [65,66]. These chaperones not only help maintain membrane stability but also suppress lipid peroxidation and protein denaturation under stress. Thus, the comprehensive downregulation of the HSP system not only reflects a diminished capacity to cope with cryo-stress but may also exacerbate structural and functional damage to membranes, ultimately contributing to the overall functional decline of spermatozoa.

### 4.2. Functional Decline from Calcium Dysregulation and Energetic Failure

Sperm cryodamage involves not only structural alterations but also profound functional and biochemical abnormalities. Membrane remodeling can trigger uncontrolled calcium influx, directly affecting key signaling pathways in sperm capacitation [67]. In this study, RGN was significantly downregulated in cryopreserved sperm. RGN maintains intracellular calcium homeostasis via negative feedback regulation, and its reduced expression suggests dysregulated calcium levels [68]. Concurrently, multiple calcium-regulating proteins showed differential expression after freezing. Downregulation of CALUB and REPS1 may exacerbate calcium dyshomeostasis by potentially promoting calcium release and delaying signal termination, respectively. In contrast, upregulation of CPNE1, ANXA11, and CIB1 could synergistically enhance the initiation, propagation, and cellular response of calcium signaling [69,70,71]. Elevated intracellular calcium further influences the cAMP/PKA pathway. We observed upregulation of adenylate cyclase (ADCY1) and downregulation of phosphodiesterase (PDE4D), which together may sustain elevated cAMP levels and thereby potentially activate protein kinase A (PKA). This triggers a signaling cascade involving the phosphorylation of serine, threonine, and tyrosine residues. While not directly measured via Western blot, the observed dysregulation of these upstream kinases and phosphodiesterases provides strong molecular evidence for premature capacitation [72,73]. These findings align with studies in common carp and Eurasian perch (*Perca fluviatilis*) [74], indicating that freeze–thaw cycling may induce spontaneous activation of sperm, depleting energy reserves required for motility, binding, and fertilization.

Sperm function is highly dependent on energy supply, and mitochondrial activity is essential for maintaining motility and fertilizing capacity [29,75]. JC-1 flow cytometry in this study revealed that the proportion of sperm with high mitochondrial membrane potential (ΔΨm) dropped from 94.48% to 28.38% after thawing. Although this value is higher than that reported in coho salmon (*Oncorhynchus kisutch*) (12.50%) [32], it is lower than that in Atlantic salmon (*Salmo salar*) (51.7–61%) [7] and yellowtail kingfish (*Seriola lalandi*) (55.4%) [76], suggesting high mitochondrial sensitivity to cryodamage. Further ELISA assays showed significantly decreased levels of T-ATP and SDH after cryopreservation, alongside a slight increase in LDH. As a key glycolytic enzyme involved in NAD^+^/NADH balance, elevated LDH often reflects cellular injury [77,78]. T-ATPase, responsible for ATP hydrolysis, is strongly linked to sperm motility, while SDH, a key TCA cycle enzyme, reflects mitochondrial functional state. Their decline indicates impaired energy metabolism. Proteomic and metabolomic data further revealed downregulation of key glycolytic enzymes (TPI1A, PGM2, PGAM1, FBP1) and metabolites (α-D-glucose, fructose-6-phosphate, pyruvate), suggesting broad suppression of glycolysis.

Omics analysis indicated that post-thaw differentially expressed molecules were mainly enriched in carbohydrate metabolic pathways, including the pentose phosphate pathway, galactose metabolism, pyruvate metabolism, and glycolysis. Hub proteins TALDO1 and GALE were implicated in coordinating metabolic balance among these pathways. While aquatic sperm primarily rely on mitochondrial respiration for ATP production [79], this study revealed significant downregulation of key TCA cycle metabolites (pyruvate, fumarate, L-malate, citrate) and the rate-limiting enzyme ACO1, which led to reduced generation of reducing equivalents (NADH, FADH_2_), ultimately impairing the electron transport chain (ETC) efficiency and ATP synthesis.

ATP production in mature sperm depends on mitochondrial oxidative phosphorylation (OXPHOS), which occurs in the cristae structure of the inner mitochondrial membrane [80]. Ultrastructural observations show that the mitochondrial cristae in cryopreserved sperm are severely damaged, likely disrupting the assembly basis of the ETC supercomplex. This structural damage was corroborated at the molecular level. Proteomic data showed significant downregulation of NDUFAF2 and SDH5 (involved in Complex I and II biogenesis) and COQ6 (which stabilizes the ubiquinone pool) in cryopreserved sperm, collectively potentially disrupting electron flow. At the same time, impaired expression of MFF and ATG7, key regulators of mitochondrial fission and autophagy, may hinder the clearance of dysfunctional mitochondria. Notably, we observed upregulation of TMEM11 and PHB2 following cryopreservation. TMEM11 and PHB2 contribute to cristae formation and inner membrane stability, respectively, and their increased expression may represent a compensatory response to structural injury [81,82]. Additionally, we found that DAMs dihydrolipoate and adenosine monophosphate were downregulated in frozen sperm. Given their important roles in energy substrate supply and signal transduction [83,84], this further suggests that the cells may be experiencing energy metabolic system collapse, directly affecting flagellar motility and membrane ion balance, and exacerbating the decline in sperm motility and function after freezing.

### 4.3. Lipid Peroxidation and Antioxidant System Deficit

During oxidative phosphorylation, electron leakage from the ETC is the primary source of ROS [80]. ROS play a dual role in sperm physiology: at low concentrations, they participate in signal transduction and regulation of capacitation, whereas excessive levels induce oxidative stress, leading to lipid peroxidation, mitochondrial dysfunction, DNA damage, and apoptosis [53]. Flow cytometry analysis in this study revealed a significant increase in ROS levels in Sichuan bream sperm after cryopreservation, consistent with observations in mammals or fish [85,86]. Given the high abundance of polyunsaturated fatty acids in the membranes of sperm from aquatic organisms, these organisms are particularly susceptible to lipid peroxidation [24]. Metabolomic analysis indicated upregulation of 5-Hete, a metabolite derived from the 5-lipoxygenase (5-LOX) pathway of arachidonic acid metabolism, suggesting activation of enzymatic lipid peroxidation and a pro-oxidative–inflammatory feedback loop [87]. Proteomic profiling further revealed dysregulation of several key proteins involved in arachidonic acid metabolism in cryopreserved sperm. Downregulation of CBR1 may impair the clearance of toxic aldehydes (e.g., 4-HNE), exacerbating secondary oxidative damage [88]. Decreased PLTP levels likely weakened the vitamin E-dependent membrane lipid antioxidant system [89]. Collectively, these results confirm significant lipid peroxidation damage in cryopreserved sperm.

Regarding the antioxidant system, the activities of SOD, CAT, and GR were significantly reduced after freezing–thawing, likely impairing the sequential clearance of O_2_^−^ and H_2_O_2_ and contributing to ROS accumulation. Consistent with these enzymatic declines, Western blot analysis further confirmed the significant downregulation of key antioxidant proteins, including SOD1, SOD2, CAT, and GPX5, providing robust evidence for the compromised antioxidant machinery at the translational level. The decline in GR activity particularly likely disrupted the balance between reduced (GSH) and oxidized (GSSG) glutathione in the glutathione cycle [90]. Furthermore, downregulation of the hub proteins G6PD and H6PD limited NADPH production, further potentially weakening GR activity and GSH regeneration capacity [91,92]. These findings highlight the central role of glutathione metabolism in cryodamage.

Integrated analysis of the glutathione metabolic pathway revealed coordinated downregulation of multiple key components in cryopreserved sperm, indicating a potential systemic collapse of the antioxidant defense system. Reduced expression of the key glutathione synthesis enzymes GCLM and GSS may contribute to markedly lower GSH levels [93,94]. Limited G6PD activity restricted NADPH supply, potentially limiting GSSG reduction and regeneration. At the antioxidant execution level, downregulation of PRDX6 likely impaired hydrogen peroxide clearance, while decreased levels of various glutathione S-transferases (GSTP1, GSTM3, GST) compromised detoxification of electrophilic compounds [94]. Metabolomic analysis showed a significant decrease in ascorbic acid (Vitamin C), potentially reducing its ROS-scavenging capacity [95]. Glutamic acid, a precursor for GSH synthesis, was also reduced, limiting GSH production. Decreased levels of protective polyamines, such as spermidine and spermine, further indicated widespread metabolic dysregulation and the potential failure of cellular protective mechanisms under oxidative stress [96].

Beyond disrupting membrane structure and mitochondrial function, lipid peroxidation can also induce DNA damage and apoptosis. Studies in various fish species have linked low hatch rates in cryopreserved sperm to high DNA fragmentation [97]. In this study, however, TUNEL flow cytometry did not detect a significant difference in DNA fragmentation levels before and after cryopreservation. Fertilization trials also showed no significant difference in hatch rates between cryopreserved and fresh sperm, suggesting that genomic stability was well-preserved despite structural and energy metabolism impairments.

Several factors may account for this stable hatching rate. First, the hatching rate was calculated relative to fertilized eggs, specifically reflecting the developmental competence of surviving embryos rather than the overall fertilization capacity of the sperm population. Second, sperm redundancy and the selective role of the narrow egg micropyle may act as a physical filter, ensuring that only physiologically superior sperm achieve fertilization [98]. Furthermore, since early fish embryogenesis is primarily driven by maternal mRNAs and proteins, embryonic development can proceed normally as long as the paternal genome is delivered without significant fragmentation [99]. This discrepancy highlights the species-specific nature of cryo-vulnerability: in Sichuan bream, the structural integrity of spermatozoa appears significantly more susceptible to cryodamage than genomic stability, aligning with findings in the Brazilian fish (*Brycon insignis*) where motility defects outweigh genetic damage [100].

We speculate that taurine, added to the cryoprotectant extender, may have mitigated DNA damage by neutralizing ROS; metabolomic data confirmed its increased levels post-thaw, consistent with its documented protective role [101,102]. However, the absence of overt DNA fragmentation does not necessarily imply a preserved epigenetic landscape. Our targeted re-analysis of the proteome revealed that cryopreservation significantly perturbed key chromatin regulatory networks. Specifically, KEGG enrichment highlighted the dysregulation of the Polycomb repressive complex and ubiquitin-mediated proteolysis, both of which are critical for maintaining chromatin compaction and histone modification integrity. At the molecular level, we observed the downregulation of Chromobox protein homolog 3 (CBX3/HP1γ), a fundamental heterochromatin organizer [103], and RBBP7, a core component of the nucleosome remodeling and deacetylase (NuRD) complex [104]. These molecular deficits provide a mechanistic basis for the reduced nuclear electron density and chromatin decondensation observed in our ultrastructural analysis. Although these epigenetic perturbations did not compromise immediate hatching rates, they may pose latent risks for long-term offspring fitness and transgenerational health—a phenomenon that warrants further investigation in future studies.

Given that fertilization in fish is an extremely rapid event occurring within minutes of activation, immediate ATP availability and the structural integrity of the motility apparatus are absolute prerequisites for sperm to traverse the micropyle. In this study, the comprehensive downregulation of glycolysis, TCA cycle, and oxidative phosphorylation pathways, coupled with severe axonemal defects, likely constitutes the primary determinant of fertilization failure. While the compromised antioxidant system and lipid peroxidation do not directly drive motility, they establish a deteriorated physiological baseline that predestines functional incompetence even before activation. Finally, it is necessary to acknowledge a technical limitation inherent to our experimental design. To satisfy the stringent purity requirements for multi-omics and biochemical analyses, sperm samples were subjected to centrifugation and washing to remove seminal plasma and cryoprotectants. Although identical low-speed processing was applied to fresh controls to offset systematic errors, we cannot entirely rule out the possibility that these procedures may have slightly exacerbated structural damage or caused partial leakage of intracellular components in frozen–thawed spermatozoa. Future studies utilizing centrifugation-free approaches would be valuable to validate these findings under conditions of minimal post-thaw manipulation.

## 5. Conclusions

This study systematically elucidates the multi-level mechanisms underlying the decline in sperm quality in Sichuan bream following cryopreservation. Our results demonstrate that the freezing–thawing process not only causes significant damage to sperm ultrastructure but also disrupts energy metabolism and impairs the oxidative defense system. Integrated multi-omics analysis further revealed that the above functional impairments were closely associated with key molecular events, including cytoskeletal remodeling, lipid metabolism reprogramming, and dysregulation of the molecular chaperone network. Our work constructed a molecular regulatory network of sperm freeze–thaw damage, deepened the understanding of the cryobiological mechanism of fish sperm, and provided a theoretical basis for developing species-specific sperm protection strategies targeting specific metabolic pathways.

## Figures and Tables

**Table 1:** Kinematic parameters of fresh and cryopreserved Sichuan bream sperm.

Parameters	Fresh Sperm	Cryopreserved Sperm
MOT (%)	82.22 ± 1.92	39.59 ± 7.81 **
VAP (μm s^−1^)	45.08 ± 2.17	13.19 ± 9.53 **
VCL (μm s^−1^)	42.49 ± 1.5	15 ± 7.55 **
VSL (μm s^−1^)	31.47 ± 1.72	6.94 ± 5.02 **
ALH (μm)	1.21 ± 0.03	0.55 ± 0.1 **
BCF (Hz)	16.48 ± 0.93	23.15 ± 1.49 **
MAD (°)	62.15 ± 2.79	117.28 ± 8.81 **
WOB (%)	102.17 ± 3.76	62.33 ± 13.76 **
LIN (%)	67.83 ± 1.07	32.08 ± 6.84 **
STR (%)	65.67 ± 1.25	42.14 ± 4.43 **

MOT: motility; VAP: average path velocity; VCL: curvilinear velocity; VSL: straight-line velocity; ALH: amplitude of lateral head displacement; BCF: beat–cross frequency; MAD: mean angular displacement; WOB: wobble (VAP/VCL); LIN: linearity (VSL/VCL); STR: straightness (VSL/VAP). ** indicates a statistically significant difference compared to fresh sperm (*p* < 0.01).

## Data Availability

The data reported in this paper have been deposited in the OMIX, China National Center for Bioinformation/Beijing Institute of Genomics, Chinese Academy of Sciences (https://ngdc.cncb.ac.cn/omix, accessed on 20 October 2025, accession no. OMIX012425 and OMIX012426).

## Supplementary Materials

The following supporting information can be downloaded at: https://www.mdpi.com/article/10.3390/ani16071014/s1, Figure S1: Overview of proteome sequencing information. (A) Distribution of peptide lengths. (B) Distribution of peptide numbers. (C) Distribution of protein molecular weights; Figure S2: OPLS-DA score plot (A) and permutation test overview (B) for different comparison group samples. Table S1: Details of differentially abundant proteins; Table S2: Statistical table of GO enrichment analysis; Table S3: Statistical table of KEGG enrichment analysis; Table S4: Metabolite annotation information; Table S5: Details of differential abundance metabolites; Table S6: KEGG enrichment results of differential abundance metabolites; Table S7: Correlation analysis of differentially abundant proteins and metabolites.

## Abbreviations

The following abbreviations are used in this manuscript:

ACO1	Aconitase 1
ACTN1	Alpha-Actinin-1
ACTR1B	Beta-Centractin
ADCY1	Adenylate Cyclase 1
AMDHD2	Amidohydrolase Domain Containing 2
ANXA11	Annexin A11
ARPC5L	Actin-Related Protein 2/3 Complex Subunit 5 Like
ATG7	Autophagy Related 7
CALUB	Calumenin b
CBR1	Carbonyl Reductase 1
CFAP300	Cilia- And Flagella-Associated Protein 300
CFL2	Cofilin 2
CIB1	Calcium And Integrin Binding 1
COQ6	Coenzyme Q6, Monooxygenase
CPNE1	Copine 1
G6PD	Glucose-6-Phosphate Dehydrogenase
GALE	UDP-galactose-4-epimerase
GMPS	Guanine Monophosphate Synthase
GSTM3	Glutathione S-Transferase Mu 3
GSTP1	Glutathione S-Transferase Pi 1
H6PD	Hexose-6-Phosphate Dehydrogenase/Glucose 1-Dehydrogenase
HSP70	Heat Shock Protein 70
HSP90A	Heat Shock Protein 90 Alpha Family Class A Member 1
HSPBP1	HSPA (Hsp70) Binding Protein 1
MFF	Mitochondrial Fission Factor
NPC2	NPC Intracellular Cholesterol Transporter 2
PCYT1A	Phosphate Cytidylyltransferase 1A, Choline
PCYT2	Phosphate Cytidylyltransferase 2, Ethanolamine
PGAM1	Phosphoglycerate Mutase 1
PGM2	Phosphoglucomutase 2
PHB2	Prohibitin 2
PLG	Plasminogen
PLTP	Phospholipid Transfer Protein
PRDX6	Peroxiredoxin 6
REPS1	RALBP1-Associated Eps Domain Containing 1
RGN	Regucalcin
RPL31	Ribosomal Protein L31
SDH5	Succinate Dehydrogenase Complex Assembly Factor 5
STK33	Serine/Threonine Kinase 33
TALDO1	Transaldolase 1
TBCE	Tubulin Folding Cofactor E
TBCD	Tubulin Folding Cofactor D
TMEM11	Transmembrane Protein 11
TPI1A	Triosephosphate Isomerase 1a
WASL	Wiskott–Aldrich Syndrome Like
WDR1	WD Repeat Domain 1
